# Sourdough “Biga” Fermentation Improves the Digestibility of Pizza Pinsa Romana: An Investigation through a Simulated Static In Vitro Model

**DOI:** 10.3390/nu15132958

**Published:** 2023-06-29

**Authors:** Alice Costantini, Michela Verni, Federica Mastrolonardo, Carlo Giuseppe Rizzello, Raffaella Di Cagno, Marco Gobbetti, Mario Breedveld, Suzan Bruggink, Kristof Lefever, Andrea Polo

**Affiliations:** 1 Faculty of Agricultural, Environmental and Food Sciences, Libera Universitá di Bolzano, Piazza Universitá 5, 39100 Bolzano, Italy; 2 Department of Environmental Biology, “Sapienza” University of Rome, Piazzale Aldo Moro 5, 00185 Rome, Italy; 3 Fourneo, 300 Rue Gilbert Chiquet, 62500 Leulinghem, Francesuzan.bruggink@fourneo.fr (S.B.);

**Keywords:** sourdough, Pinsa Romana, protein quality, in vitro digestion, starch digestibility, indirect process

## Abstract

Baked goods manufacturing parameters and fermentation conditions interfere with the nutrients content and affect their gastrointestinal fate. Pinsa Romana is a type of pizza that, recently, has been commercially rediscovered and that needed elucidation from a nutritional and digestibility perspective. In this study, six types of Pinsa Romana (five made with indirect method and one produced with straight dough technology) were characterized for their biochemical and nutritional features. Several variables like indirect (biga) Pinsa Romana production process, fermentation time and use of sourdough were investigated. The Pinsa Romana made with biga including sourdough and fermented for 48 h at 16 °C ((PR_48(SD)) resulted in the lowest predicted glycemic index, in the highest content of total peptides, total and individual free amino acids and gamma-amino butyric acid (GABA), and in the best protein quality indexes (protein efficiency ratio and nutritional index). The static in vitro digestion showed that the digesta from PR_48(SD) confirmed a reduced in vitro glycemic response after intake, and it showed a lower bioavailability of hydrophilic peptides. Furthermore, the inclusion of sourdough in biga enhanced the bioavailability of protein-related end-products including human health promoting compounds such as essential amino acids.

## 1. Introduction

Baked goods refer to a wide range of food with different textures and flavors that can be designed to provide a variety of nutritional properties [1,2]. In particular, the sensory and nutritional features of bakery products are the result of a thoughtful choice of manufacturing processes and production procedures [3]. In recent years, the so called “Pinsa” (from the Latin “pinsere”; to stretch) Romana (PR), a type of elongated pizza made with a simple recipe including flours (e.g., wheat, soy, and rice), oil, leavening agent (baker’s yeast or sourdough), salt, and a high water content that ensure the crunchiness of the crust and the softness of the crumb, was commercially rediscovered [4]. PR has already been investigated for the influence of combined flours (einkorn, rice, and soy) on dough rheological performance [4], but the literature is lacking investigations about its nutritional features. In addition, no study has assessed the effect of different fermentation methods (like an indirect fermentation process, and the inclusion of sourdough in the yeasted pre-ferment) on the nutritional benefits and in vitro digestibility.

The production process of PR requires a long leavening, commonly from 24 to 72 h [4]. It follows that the fermentation process and the leavening agent, such as baker’s yeast or sourdough, are of utmost importance for PR manufacturing [5]. Baker’s yeast is the most widely adopted starter for industrial productions and it can be used directly (straight dough technology) and mixed with the other ingredients into the final dough, or indirectly (indirect method) to prepare an intermediate pre-ferment to be added in the final dough [5,6]. Biga is a type of leavening agent originally used as a method of stiffening dough in Italian bakeries. The use of biga gives the final product a more complex flavor, better texture, and increased shelf-life in comparison with the direct method [6]. On the other hand, while baker’s yeast represents a faster solution, sourdough is one of the oldest natural leavening starters that are more frequently used for artisanal productions. The features that differentiate the two starters are thoroughly proven: sourdough fermentation has the power to decrease the content of anti-nutritional factors (like phytic acid), toxins and allergens, and the glycemic index, while increasing the biodiversity of peptides, free amino acids (FAA) and proteins [7,8]. Moreover, sourdough fermentation improves the digestibility and the release of health-promoting compounds that can be more available during the digestive process at the gastrointestinal (GI) level [9,10,11,12,13].

In this context, food absorption, protein and starch digestibility depend on mechanical, chemical, and biochemical events that allow the disruption of nutrients and bioactive compounds [14]. As a result of this complex mechanism, the biodiversity of the end-products from GI degradation of different baked goods still needs further elucidation. Indeed, within a global “foodomics” perspective, the monitoring of food protein derivatives at the GI level can highlight functional compounds with health-promoting activity, as well as compounds that may have adverse effects on human health (i.e., allergies, and intolerances) [15]. Enzymatic proteolysis can occur during GI digestion, and/or through laboratory procedures (proteolytic enzymes) or certain food processing (e.g., fermentation and cooking). During this process, a large portfolio of bioactive peptides with antidiabetic, cholesterol-lowering, antihypertensive, anticancer, antimicrobial and multifunctional properties is released [16]. As a result of enzymatic proteolytic activity, amino acids are important natural compounds for GI health [17]. In addition, understanding the mechanism of starch hydrolysis during digestion and the subsequent generation of simple sugars is an important factor to predict physiological glycemic postprandial effects of food intake [18,19]. In this sense, an investigation of the GI fate of carbohydrates would enrich the knowledge about the glycemic index and potential properties of leavened baked goods. Facing this background, the in vitro digestion procedures can be considered valuable tools to investigate potential health-related benefits associated with an improved digestibility of a wide range of different bakery products [20].

First, our study aimed at assessing how different PR-making processes (direct or indirect) affect nutritional properties and digestibility. Additionally, the combined use of biga and sourdough, and the length of the fermentation stage in the yeasted biga were assessed to highlight the conditions that affect the digestibility and the overall quality of the PR. To that end, the static INFOGEST in vitro digestion method [21] and static dialysis [10] were adopted to simulate the digestion conditions occurring in the human digestive tract after the PR consumption, considering realistic transit time, pH, and enzymatic conditions for every digestion step [21,22]. The specific protein and starch degradation was monitored on digested samples opening a window into the digestibility of PR made through different processes and plausible underlying mechanisms.

## 2. Materials and Methods

### 2.1. Raw Materials

Commercial soft wheat *(Triticum aestivum* L.) flour (Grands Moulins de Paris, Ivry sur Seine, Ile-de-France, France), stone ground wheat (*Triticum aestivum* L.) flour (Grands Moulins de Paris, Ivry sur Seine, Ile-de-France, France), rice (*Oryza sativa* L.) flour (Inveja, Twello, The Netherlands) and soy (*Glycine max* L. (Merr.)) flour (Herba Ingredients, Wormer, The Netherlands) were used. The gross chemical composition of the flours used for making sourdoughs, biga and PRs were as follows: soft wheat flour, protein 12.1 g/100 g, fats 1.1 g/100 g, carbohydrates 67.1 g/100 g, fibers 4.0 g/100 g; stone ground wheat flour, protein 10.7 g/100 g, fats 1.5 g/100 g, carbohydrates 67.0 g/100 g, fibers 5.3 g/100 g; toasted full fat soy flour, protein 40.5 g/100 g, fat 20.8 g/100 g, carbohydrates 10.6 g/100 g, fibers 14.5 g/100 g; rice flour, protein 8.3 g/100 g, fats < 1.5 g/100 g, carbohydrates 76.0 g/100 g, fiber 1.0 g/100 g.

### 2.2. Sourdough Preparation

A spontaneous mother dough was prepared by mixing 50% of stone ground wheat flour and 50% of tap water and fermented for 18 h at 30 °C. The procedure was repeated five times until the mother dough was stable, and it was continuously subjected to a maintenance process (DY = 200; 4 h at 30 °C). The final sourdough (SD) was prepared by mixing soft wheat flour, tap water and the stable mother dough at the ratio of 37.5%, 37.5% and 25%, respectively, and it was used for the production of biga and PRs. The SD was mature after fermenting for 4 h at 30 °C. Before use, SD was kept for 8 h at 4 °C.

### 2.3. Biga and PRs Making

**Figure 1:**
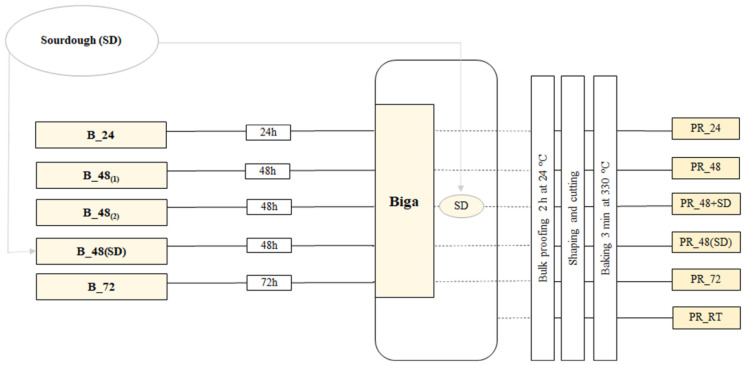
Processflow of six different Pinsa Romana used in the study. B_24, biga fermented for 24 h with baker’s yeast; B_48_(1)_, biga fermented for 48 h with baker’s yeast; B_48_(2)_, biga fermented for 48 h with baker’s yeast; B_72, biga fermented for 72 h with baker’s yeast; B_48(SD), biga fermented for 48 h with baker’s yeast and sourdough. PR_24, Pinsa Romana made with biga fermented for 24 h with baker’s yeast; PR_48, Pinsa Romana made with biga fermented for 48 h with baker’s yeast; PR_72, Pinsa Romana made with biga fermented for 72 h with baker’s yeast; PR_48(SD), Pinsa Romana made with biga fermented for 48 h with baker’s yeast and sourdough; PR_48+SD, Pinsa Romana made with biga fermented for 48 h with baker’s yeast, and including sourdough in the final dough. PR_RT, Pinsa Romana made without biga and sourdough (control sample). SD means sourdough.

Five types of biga that differ in the time of fermentation (24, 48 or 72 h) and/or addition of SD were prepared. The temperature of fermentation was 16 °C for all types of biga. When SD was included in the recipe, it was added at 5% (*w*/*w*) and the sourdough-biga was fermented for 48 h. The different biga were used to produce five different PRs as detailed in Table S1. Specifically, three kinds of PRs were manufactured with the same recipe (i.e., water, (10%, *w*/*w*), olive oil (1.5%, *w*/*w*) and salt (1%, *w*/*w*)) and addition of the baker’s yeast biga fermented for 24 or 48 or 72 h (PR_24, PR_48 and PR_72, respectively). One PR was produced with the same raw ingredients and the addition of both the baker’s yeast-biga fermented for 48 h and 19% (*w*/*w*) of SD (PR_48+SD). The last PR was manufactured with the same raw ingredients and the addition of the sourdough-biga (PR_48(SD)). A control thesis was also produced with the same raw materials but without the use of either biga or SD (PR_RT). Table 1 summarizes the formulations used for biga and PRs manufactured for the study. The six PR doughs were leavened for 2 h at 24 °C followed by 1.5 h processing and resting. PRs were baked at 330 °C for 3 min. The workflow of the entire process is shown in Figure 1.

### 2.4. Physical and Microbiological Characterization of Biga and SD

SD and bigas pH was determined through a pH meter (Model 507, Crison, Milan, Italy) with a food penetration probe. To assess the cell density of lactic acid bacteria (LAB) and yeast, ten grams of each biga or SD were added to 90 mL of physiological solution (NaCl, 0.9% *w*/*v*) and homogenized with a Classic Blender (PBI International, Milan, Italy) to obtain a mother suspension. Decimal serial dilutions were prepared in 9 mL of sterile physiological solution. LAB were enumerated through the plate count method after the incubation at 30 °C for 48 h using modified MRS agar (Oxoid, Basingstoke, Hampshire, UK) medium, prepared by the addition of 0.5% (*w*/*v*) maltose and 0.5% (*w*/*v*) yeast extract to standard MRS medium and supplemented with cycloheximide (0.1 g/L) (Sigma-Aldrich, St. Louis, MO, USA). Yeasts cell density was estimated on Sabouraud dextrose agar (Oxoid, Basingstoke, Hampshire, UK) medium supplemented with chloramphenicol (0.1 g/L) (Sigma-Aldrich, USA) after incubation at 30 °C for 48 h. A control dough produced without biga and SD was also considered for such characterizations. After growth, colonies from SD with different morphologies were isolated from the highest plate dilution and identified by the partial sequencing of the gene encoding for 16S rRNA (for LAB) and for 26S rRNA (for yeasts) as reported by [23].

### 2.5. In Vitro Static Digestion Procedure

**Figure 2:**
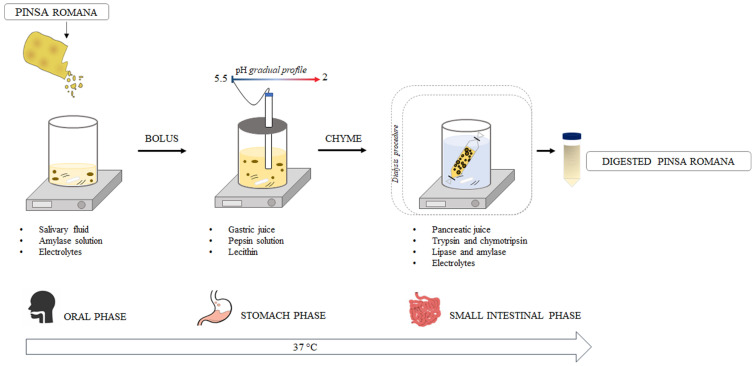
Graphical representation of the in vitro simulation of upper gastrointestinal tract transit and digestion.

The in vitro digestion of different PRs (PR_24, PR_48, PR_48(SD), PR_48+SD, PR_72 and PR_RT) was carried out according to the consensus method developed within a large European framework (COST Action InfoGest) with some modifications [21,24,25]. Figure 2 describes the steps carried out along all the digestion procedures.

They include an initial oral phase in which PR samples were freeze-dried, ground, and diluted 1:2 (dry, *w*/*w*) with simulated salivary fluid (SSF, pH 7) solution (KCl, 15.10 mM; KH_2_PO_4_, 3.70 mM; NaHCO_3_, 13.60 mM; MgCl_2_(H_2_O)_2_, 0.15 mM; (NH_4_)_2_CO_3_, 0.06 mM). The suspension was added with 0.75 mL of amylase solution (1500 U/mL of SSF) and CaCl_2_ 0.3 M solution (41.98 μL). The final mixture was mixed for 2 min at 37 °C. Then a stomach phase followed in which the oral bolus was diluted with a mixture mimicking the gastric content. The stomach phase incubation started by adding 82.02 mL of gastric juice (0.04 M KCl and 0.24 M NaCl). The suspension was supplemented with 6.07 mL of 2% pepsin solution (≥400 U/mg) and 0.68 mL of lecithin solution (0.17 mM). The incubation lasted for 2 h at 37 °C, with a pH gradient from 5.5 to 2.0. Finally, a small intestinal phase took place in which the gastric chyme (140 mL) was added with 50.00 mL of simulated pancreatic juice (NaHCO_3_ 0.19 M and 8 g/L of Oxgall (Difco™ Oxgall, BD, Franklin Lakes, NJ, USA)), 4.5 mL of trypsin solution (50 mg of trypsin 5000 USP-U/mg in 5 mL of 1 mM HCl), 5.6 mL of chymotrypsin (100 mg chymotrypsin ≥ 1000 USP-U/mg in 10 mL of 1 mM HCl), 180 mg of lipase (type II, 100–500 U/mg) and 300 mg of hog α-amylase (50 U/mg), and 350 μL of 0.3 M CaCl_2_. The entire mixture (200 mL) was incubated for a further 3 h at 37 °C under static dialysis with a 14 kDa membrane in 400 mL of 0.04 M NaHCO_3_ dialysis solution (pH 7) within a bioreactor, according to [10]. After the incubation, the digesta was collected and stored at −80 °C until further analyses. The prefix “D-“was used to identify the digesta from six different PRs: D-PR_24, D-PR_48, D-PR_48(SD), D-PR_48+SD, D-PR_72 and D-PR_RT.

### 2.6. Biochemical and Nutritional Characterization of the PRs

#### 2.6.1. Nutritional Label

Moisture, ash, protein (total nitrogen × 5.7) and fats of different PRs were determined according to ISTISAN Report 96/34 [26]. Total carbohydrates were calculated as:

(1) Total carbohydrates=100−(proteins+lipids+ash)

Proteins, lipids, carbohydrates, and ash were expressed as % of fresh weight (fw).

#### 2.6.2. pH, Total Treatable Acidity (TTA) and Phytic Acid

The pH of different PRs was determined as mentioned in paragraph 2.4. Total titratable acidity (TTA) was also measured. Briefly, 10 g of the six PRs was homogenized with 90 mL of distilled water and TTA was expressed as the amount (mL) of 0.1 M sodium hydroxide (NaOH) required to neutralize the solution (pH, 8.3) according to the official AACC method 02–31.01 [27]. Phytic acid content was determined using the Megazyme phytic acid phytate/total phosphorous K-PHIT kit (Megazyme International, Wicklow, Ireland).

#### 2.6.3. In Vitro Protein Digestibility (IVPD), Nutritional Indexes and Predicted Glycemic Index (pGI)

The evaluation of IVPD was carried out on PR_24, PR_48, PR_48(SD), PR_48+SD, PR_72 and PR_RT according to the method of Akeson and Stahmann [28], with some modifications [29]. The resulting supernatant containing the digested fraction, was used for the estimation of proteins [30], peptides [31], and FAA [32]. IVPD was expressed as a ratio between digested fraction and total protein [IVPD = (digested protein/total protein) × 100].

The digested protein fraction, deriving from 1 g of PR_24, PR_48, PR_48(SD), PR_48+SD, PR_72 and PR_RT samples, was added to 5.7 M HCl, under a nitrogen steam and incubated at 110 °C for 24 h. After freeze-drying, the hydrolysate was resuspended in lithium citrate buffer, pH 2.2, and filtered through a Millex-HA 0.22 µm pore size filter (Millipore Co., Burlington, MA, USA). The modified AOAC method (2005) [33] was used to determine the total amino acid profile. Amino acids, analyzed by a Biochrom 30+ series Amino Acid Analyzer, were used to determine nutritional indexes. Tryptophan content was estimated with the [34] method.

The Essential Amino Acid (EAA) index, which estimates the quality of the test protein, using its EAA content as a criterion [35], was calculated according to the equation:

(2) $$\mathrm{EAA}\;\mathrm{index}=\sqrt{\frac{\left({\mathrm{EAA}}_{1}\times 100\right)\left({\mathrm{EAA}}_{2}\times 100\right)\left(\ldots \right)\left({\mathrm{EAA}}_{n}\times 100\right)\left[\mathrm{sample}\right]}{\left({\mathrm{EAA}}_{1}\times 100\right)\left({\mathrm{EAA}}_{2}\times 100\right)\left(\ldots \right)\left({\mathrm{EAA}}_{n}\times 100\right)\left[\mathrm{reference}\right]}}$$

The Biological Value (BV) indicates the utilizable fraction of the test protein [35], and was calculated using the equation:

(3) BV =([1.09×EAA index]−11.70)

The Protein Efficiency Ratio (PER), which estimates the protein nutritional quality based on the amino acid profile after hydrolysis, was determined using the equation developed by Ihekoronye [36]:

(4) PER =−0.468+(0.454×[Leucine])−(0.105×[Tyrosine])

The Nutritional Index (NI) normalizes the qualitative and quantitative variations of the test protein compared to its nutritional status. NI was calculated using Crisan and Sands equation [37] which considers all the factors with equal importance:

(5) NI =(EAA×Protein (g/100 g)×100)

The sequence of limiting EAA, corresponding to the list of EAA having the lowest Chemical Score (CS), which estimates the amount of protein required to provide the minimal EAA pattern for adults, was calculated using the equation of Block and Mitchell [38].

The degree of starch digestion was expressed as a percentage of potentially available hydrolyzed starch according to [9]. The pGI of different PRs was determined through quantification of the hydrolysis index (HI) according to [39] using the equation previously proposed by [40]. A reference sample (white bread) was used as a control to estimate the hydrolysis index (HI = 100).

### 2.7. Biochemical and Nutritional Analysis before and after Small Intestinal In Vitro Digestion of PRs

#### 2.7.1. Organic Acids, Sugars, and Dietary Fibers

Water/salt-soluble extracts (WSE) from PR_24, PR_48, PR_48(SD), PR_48+SD, PR_72 and PR_RT were prepared according to [41]. Lactic and acetic acids, glucose, fructose, maltose, and mannitol were determined on WSE and on the liquid-phase of the digested PRs using an Ultimate 3000 high-performance liquid chromatography (Thermo Fisher Scientific, MA, USA) equipped with an Aminex HPX-87H column (300 mm × 7.8 mm, 9 µm) (ion exclusion, Bio-Rad, Hercules, CA, USA) [42]. Lactic and acetic acids were determined through a UV detector operating at 210 nm, while glucose, fructose, maltose, and mannitol were detected by a 200a refractive index detector (Perkin Elmer, Waltham, MA, USA). The total dietary fibers and the content of resistant starch were determined both on PR_24, PR_48, PR_48(SD), PR_48+SD, PR_72 and PR_RT and on their digested counterparts (D-PR_24, D-PR_48, D-PR_48(SD), D-PR_48+SD, D-PR_72 and D-PR_RT). Dietary fibers were determined through the AOAC Official Method 991.43 [33]. The resistant starch content was evaluated using K-RSTAR (Megazyme Ltd., Wicklow, Ireland) based on the method described by AOAC 2002.02 [43].

#### 2.7.2. Total and Free Individual Amino Acids, Total Peptides and Determination of Peptide Profiles by Reversed-Phase Fast Performance Liquid Chromatography (RP-FPLC)

For FAA analysis the WSE of PRs and the liquid-phase of the corresponding digesta were used with a Biochrom 30^+^ series Automatic Amino Acid Analyzer (Biochrom Ltd., Cambridge Science Park, UK), equipped with a Li-cation-exchange column (4.6 × 200 mm internal diameter), as described by [44].

The concentration of peptides was determined both in the WSE from the PRs (PR_24, PR_48, PR_48(SD), PR_48+SD, PR_72 and PR_RT) and in liquid-phase of the corresponding digesta (D-PR_24, D-PR_48, D-PR_48(SD), D-PR_48+SD, D-PR_72 and D-PR_RT) using *o*-phthaldialdehyde (OPA) assay [31]. In addition, WSE and digesta soluble fraction were subjected to an RP-FPLC, using a Resource RPC column and an ÄKTA FPLC equipment with the UV detector at 214 nm (GE Healthcare Bio-Sciences AB, Uppsala, Sweden). The aliquots were added with 0.05% (*v*/*v*) trifluoroacetic acid (TFA) and centrifuged at 10,000× *g* for 10 min, and the supernatant was filtered through a 0.22 μm pore size filter and loaded into the column. The elution process was carried out by increasing the acetonitrile (CH_3_CN) concentration linearly from 5% to 46% between 16 and 62 min, and from 46% to 100% between 62 and 72 min at the flow rate of 1 mL/min using a mobile phase composed of water and CH_3_CN containing 0.05% TFA [29].

### 2.8. Statistical Analysis

Data used for statistical elaboration were the means of three independent analyses ± standard deviations (*n* = 3). Data were subjected to Principal Component Analysis (PCA), and to one-way ANOVA; a comparison of sample means was obtained by Tukey’s procedure with a 95% confidence interval (*p* < 0.05). The FAA overall dataset was used to generate a graphic output (pseudo-heatmap) to assess the clustering of samples from the single variable’s matrix. Statistical analysis was performed on Statistica 8.0 (StatSoft Inc., Tulsa, OK, USA) and XLSTAT^®^ software (version 2020.5.1.1052, Addinsoft, New York, NY, USA).

## 3. Results

### 3.1. Characterization of Sourdough and Biga

In the biga prepared with sourdough, after 48 h of fermentation at 16 °C, pH and LAB cell density were 3.94 ± 0.01 and 8.89 ± 0.13 Log CFU/g, respectively. The cell density of LAB increased also in biga made with the only use of baker’s yeast when it was fermented for 72 h at 16 °C (7.91 ± 0.06 Log CFU/g), while the same biga fermented for 24 h and 48 h resulted in 6.40 ± 0.21 and 6.35 ± 0.17 Log CFU/g, respectively. The cell density of LAB in the control dough produced without biga and sourdough to produce PR_RT was <5 Log CFU/g. SD (pH; 3.78 ± 0.03) used for making PR_48(SD) and PR_48+SD, had a LAB and yeasts cell density of 8.06 ± 0.01 and 7.33 ± 0.13 Log CFU/g, respectively (Table S2). Seven isolates belonging to *Fructilactobacillus sanfranciscensis* and nine isolates belonging to *Saccharomyces cerevisiae* were identified in the SD.

### 3.2. Nutritional Label of PRs

The nutritional labels of the six different PRs are reported in Table 2.

The macronutrient content showed no marked variations between the PRs analyzed. Specifically, total fat content ranged from 5.4 to 5.9 g/100 g, total carbohydrates ranged from 75.8 to 77.4 g/100 g and total protein content was between 14.8 and 16.0 g/100 g. Even slightly, PR_48(SD) showed the lowest total fat content (5.4 g/100 g) and the highest total carbohydrate concentration (77.4 g/100 g), while PR_48+SD showed the highest total fat content (5.9 g/100 g). Both PRs produced with SD showed the lowest total protein amount (14.8 g/100 g). The consumption of 100 g of PRs corresponded to an intake between 406 (PR_48) and 417 (PR_48+SD) Kcal.

### 3.3. Biochemical and Nutritional Characteristics of PRs

#### 3.3.1. pH, TTA and Phytic Acid

The combination of wheat, soy, and rice flour blend along with the use of baker’s yeast and SD in the biga samples affected the acidity of biga-containing samples and PR_48(SD) reached the lowest pH value (4.27 ± 0.02, *p* < 0.05) among all samples (Table 3).

Lower acidification (*p* < 0.05) compared to PR_48(SD) was found when SD was included only in the final dough (PR_48+SD, pH = 4.90 ± 0.02). PRs made with biga including only baker’s yeast (PR_24, PR_48, PR_72) showed a pH value significantly (*p* < 0.05) higher compared to that of those produced with SD (PR_48(SD) and PR_48+SD). As expected, in these PRs, the longer fermentation time (PR_48 and PR_72) resulted in lower pH (*p* < 0.05) compared to PR_24. The highest (*p* < 0.05) values of pH were found for the PR made without biga and sourdough (PR_RT) together with PR_24 (5.60 ± 0.03 and 5.61 ± 0.01, respectively). Accordingly, TTA values showed an opposite trend (Table 3). Phytic acid content was not affected by the different production processes (Table 3).

#### 3.3.2. IVPD, pGI and Nutritional Indexes

The recipe and the processing conditions did not affect the percentage of IVPD of the leavened baked goods, while the PR prepared with the addition of SD in the biga (PR_48(SD)) was the only one with significant (*p* < 0.05) lower pGI (63.9 ± 0.1) (Table 3). The protein quality-related nutritional indexes (EAA index, BV index, PER, NI) and their limiting amino acids are listed in Table 4. Generally, the sequence of limiting amino acids varied among samples, whereas no statistical (*p* > 0.05) differences were found in EAA and BV indexes among PRs, ranging from 68.97 ± 3.73 to 75.85 ± 5.70 and from 63.48 ± 4.11 to 70.98 ± 3.98, respectively. The inclusion of SD in biga led to a 58.1% increase (*p* < 0.05) in the PER index in PR_48(SD) compared to PR_RT. Accordingly, the NI was significantly higher (*p* < 0.05) in PR_48(SD) and PR_72 (3.87 ± 0.27 and 3.60 ± 0.17, respectively).

### 3.4. Organic Acids before and after Small Intestinal In Vitro Digestion of PRs

The inclusion of SD in PR_48(SD) and PR_48+SD favored lactic and acetic acid production (Table 5).

PR_48(SD) showed the highest (*p* < 0.05) concentration of lactic and acetic acids (102.1 ± 0.1 and 27.0 ± 0.1 mM, respectively) followed by PR_48+SD, where the SD was included only in the final dough (35.7 ± 0.1 and 14.7 ± 0.3 mM, respectively). The other PRs showed lactic acid concentrations ranging from 2.3 to 2.9 mM with no statistical (*p* > 0.05) differences. Contrarily, acetic acid was not found in PR_24 and PR_48, while PR_72 contained a low amount (1.2 ± 0.3 mM).

The digesta from PRs produced with biga including SD (D-PR_48(SD)) showed a higher (*p* < 0.05) content of lactic acid compared to D-PR_48+SD (42.0 ± 3.0 and 18.7 ± 0.6 mM, respectively), while in the digesta from PR_24, PR_48, PR_72 and PR_RT lactic acid was under the detection limit. Acetic acid was not found in the digesta from all PRs.

### 3.5. Carbohydrates before and after Small Intestinal In Vitro Digestion of PRs

The use of SD both in biga (PR_48(SD)) and in the final dough (PR_48+SD), and the longer fermentation time (PR_72) resulted in higher (*p* < 0.05) glucose concentration (32.7 ± 0.1, 17.6 ± 0.1 and 21.1 ± 0.1 mM, respectively) compared to PR_48 and PR_24. On the contrary, PR_RT, made without biga or sourdough, showed the lowest (*p* < 0.05) content of glucose (Table 5). Fructose concentration was significantly (*p* < 0.05) lower in PR_48(SD) (2.3 ± 0.1 mM) and PR_48+SD (4.5 ± 0.1 mM), and significantly (*p* < 0.05) higher in PR_72 (14.0 ± 0.1 mM) compared to other PRs. Mannitol was found only in the PRs produced with SD. However, in PR_48(SD) the concentration (41.8 ± 0.1 mM) was significantly (*p* < 0.05) higher compared to PR_48+SD (6.2 ± 0.1 mM). Furthermore, maltose was present in all PRs, with the highest (*p* < 0.05) values in PR_RT (74.7 ± 0.1 mM), followed by PR_72 (61.0 ± 0.1 mM) and PR_48(SD) (60.6 ± 0.1 mM) (Table 5).

Carbohydrates were evaluated also in the digesta from PRs to assess their fate along the GI tract. After the in vitro small intestinal digestive (D) simulation, the level of simple carbohydrates strongly changed. The fructose concentration in D-PR_48, D-PR_48(SD) and D-PR_48+SD was under the limit of quantification (< LOQ). Accordingly, after in vitro small intestinal digestion the concentration of mannitol showed the same trend already observed in PR_48(SD) and PR_48+SD. Indeed, the amount of mannitol in D-PR_48(SD) was statistically (*p* < 0.05) different compared to D-PR_48+SD (21.0 ± 2.1 and 4.4 ± 2.3 mM, respectively). Moreover, the different manufacturing parameters of PRs did not interfere (*p* > 0.05) with the maltose content in the PRs digesta that ranged from 741.2 ± 15.2 to 665.8 ± 41.4 mM. The glucose content in D-PR_48(SD) was significantly higher (*p* < 0.05) compared to D-PR_RT and D-PR_48. The glucose level in digesta was 188.5 ± 3.3 and 126.9 ± 22.7 mM in D-PR_48(SD) and D-PR_RT, respectively.

Total dietary fiber content was assessed to evaluate the behavior of non-digestible compounds through in vitro digestion. The value of total dietary fibers before digestion ranged from 15.4 ± 1.7 to 13.7 ± 0.7% d.m., while after digestion it ranged from 16.6 ± 1.1 to 13.4 ± 0.9% d.m., with no statistical (*p* > 0.05) differences among the six PRs, as well as for the digested PRs.

Resistant starch in no-digested PRs ranged from 0.7 ± 0.1 (PR_48+SD) to 1.1 ± 0.1 g/100 g d.w. (PR_24) and only PR_24 and PR_48+SD differ significantly (*p* < 0.05) (Table 5). No statistical differences (*p* > 0.05) were found in the content of RS after the digestion of PRs.

### 3.6. Protein Hydrolysis before and after Small Intestinal In Vitro Digestion of PRs

#### 3.6.1. Total Peptides and RP-FPLC Peptide Profiles

Overall, the inclusion of SD in biga resulted in the highest level of protein hydrolysis. Specifically, total peptides had higher (*p* < 0.05) abundance in PR_48(SD) (12.7 ± 0.2 g/Kg) compared to PR_48+SD (12.0 ± 0.1 g/Kg) and PR_72 (11.4 ± 0.1 g/Kg), while PR_24, PR_48 and PR_RT had the lowest (*p* < 0.05) content (Table 5). Instead, after PRs digestion, D-PR_48(SD) resulted in a similar (*p* > 0.05) total peptide amount to D-PR_48+SD (201.2 ± 8.1 and 183.1 ± 1.5 g/Kg, respectively), while the value was higher (*p* < 0.05) than D-PR_72 (165.8 ± 1.3 g/Kg).

The RP-FPLC peptide profiles of PRs and their digesta are expressed as the % of the total area (mAU·min) at different elution steps, reported as the concentration of CH_3_CN (CH_3_CN < 19%; 19% < CH_3_CN < 41%; CH_3_CN > 41%) (Table 6).

In no-digested PRs, the analysis showed a total area under the curve (AUC) from 2196.9 mAU·min to 3693.0 mAU·min (PR_RT and PR_48(SD), respectively). In the more hydrophilic zone (CH_3_CN < 19%), the highest relative values were 22.6% for PR_72 and 19.1% for PR_48(SD), while the relative lowest value was 5.8% for PR_48+SD. Conversely, increasing the CH_3_CN gradient (19% < CH_3_CN < 41%) the relative highest release of peptides was found in PR_48+SD (94.2%). No RP-FPLC peptide profiles were detected when the gradient of CH_3_CN was higher than 41% for all PRs. The analysis showed the opposite behavior in the digesta. In particular, the AUC of D-PR_RT (3611.1 mAU·min) was relatively higher than those found for the other digested PRs, followed by the digesta of PRs manufactured with longer fermented biga produced with only baker’s yeast (3518.8 and 3594.8 mAU·min for D-PR_72 and D-PR_48, respectively). The D-PR_48(SD) showed the lowest relative percentage of the total area in the hydrophilic zone, whereas it displayed the highest value (28.9%) in the elution interval from 19 to 41% of the CH_3_CN gradient. Differently from no-digested PRs, all digested PRs showed the presence of a peptides profile in the more hydrophobic zone (CH_3_CN > 41%).

#### 3.6.2. Release of FAA

All individual FAA profiles and the total content before (mg/Kg) and after (mg/L) in vitro small intestinal digestion are reported in Table S3. Before digestion, total FAA concentration (expressed as the sum of FAA and their derivatives) in PR_48(SD) was significantly (*p* < 0.05) higher (557.14 ± 14.72 mg/kg) than other PRs, followed by PR_72 (425.56 ± 11.29 mg/kg). In general, most EAA resulted higher in the PRs produced with SD or longer fermentation time (PR_48(SD), PR_48+SD and PR_72). In particular, the concentration of Val, Met, Ile, Leu, Phe, and Trp were statistically (*p* < 0.05) higher in PR_48(SD). Accordingly, the content of γ-aminobutyric acid (GABA) was the highest (*p* < 0.05) in PR_48(SD) (66.92 ± 0.97 mg/kg), followed by PR_72 (59.67 ± 0.87 mg/kg).

After digestion, D-PR_48(SD) (1275.30 ± 51.92 mg/L) and D-PR_48+SD (1205.88 ± 81.57 mg/L) showed the highest (*p* < 0.05) total FAA, followed by D-PR_72 (1068.14 ± 26.98 mg/L). Anyway, the total FAA amount in D-PR_48(SD) was ca. 19.4% higher than D-PR_72. Overall, EAA (Thr, Val, Met, Ile, Leu, Phe, His and Trp) on digesta were significantly (*p* < 0.05) higher in D-PR_48(SD), D-PR_48+SD and D-PR_72 compared to the other samples. On the contrary, D-PR_48, D-PR_24 and the digesta of the control sample (D-PR_RT) showed the lowest content for individual FAA.

**Figure 3:**
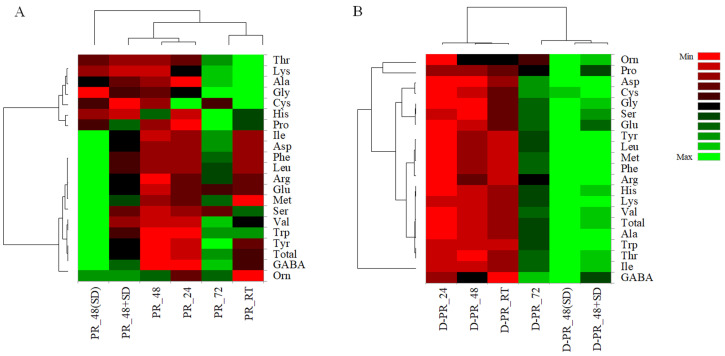
Pseudo-heatmap showing free amino acids content in Pinsa Romana before (**A**) and after (**B**) small intestinal in vitro digestion. PR_24, Pinsa Romana made with biga fermented for 24 h with baker’s yeast; PR_48, Pinsa Romana made with biga fermented for 48 h with baker’s yeast; PR_72, Pinsa Romana made with biga fermented for 72 h with baker’s yeast; PR_48(SD), Pinsa Romana made with biga fermented for 48 h with baker’s yeast and sourdough; PR_48+SD, Pinsa Romana made with biga fermented for 48 h with baker’s yeast, and including sourdough in the final dough. PR_RT, Pinsa Romana made without biga and sourdough (control sample). “D-” prefix means digested. Details about the formulation of all PR samples are described in materials and methods.

Changes in FAA content of different PRs and digested counterparts were also visualized using a pseudo-heatmap (Figure 3).

Before digestion (Figure 3A), PR_48(SD) graphically stood out for the largest number of individual FAA, forming a different cluster from the other PRs. PR_48(SD) and PR_RT showed opposite FAA profiles. In the digesta, PRs produced with SD (D-PR_48(SD) and D-PR_48+SD) or with longer fermentation (D-PR_72) grouped in a separate cluster compared to D-PR_24, D-PR_48 and D-PR_RT (Figure 3B).

### 3.7. Principal Component Analysis (PCA) on PR Common Variables before and after Small Intestinal In Vitro Digestion

**Figure 4:**
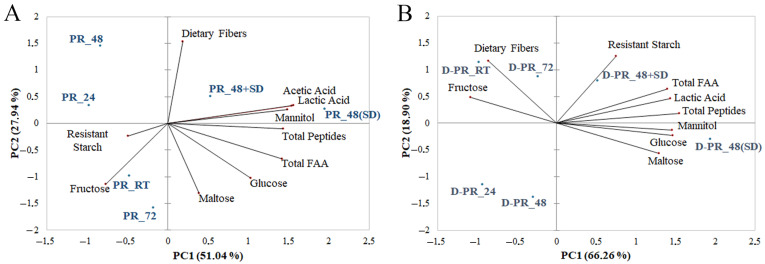
Principal Component Analysis (PCA) of the biochemical and nutritional characteristics of six Pinsa Romana before (**A**) and after (**B**) in vitro small intestinal digestion. Biplots include selected variables, sugars, lactic and acetic acids, total peptides, total free amino acids (FAA), total dietary fibers and resistant starch. PR_24, Pinsa Romana made with biga fermented for 24 h with baker’s yeast; PR_48, Pinsa Romana made with biga fermented for 48 h with baker’s yeast; PR_72, Pinsa Romana made with biga fermented for 72 h with baker’s yeast; PR_48(SD), Pinsa Romana made with biga fermented for 48 h with baker’s yeast and sourdough; PR_48+SD, Pinsa Romana made with biga fermented for 48 h with baker’s yeast, and including sourdough in the final dough. PR_RT, Pinsa Romana made without biga and sourdough (control sample). “D-” prefix means digested.

PCA was carried out to reduce the dimensionality of the data matrix both for PRs and the digested counterpart after the small intestinal in vitro digestion (Figure 4).

Selected variables (sugars, organic acids, peptides, total FAA, resistant starch and total dietary fibers) were used to differentiate the six PR samples. Before digestion, PC1 and PC2 explained 51.04% and 27.94%, respectively, of the total variance (Figure 4A). PCA clustered the samples into two groups. PRs made without the use of SD were distributed on the II and III quadrants of the plot and were characterized by the variables of resistant starch and fructose. PR_48(SD) and PR_48+SD were grouped in the opposite zone of the plane and were differentiated for acetic acid, lactic acid, mannitol, and total peptides. After the in vitro small intestinal digestion, PC1 and PC2 described 66.26% and 18.90% of the total variance, respectively (Figure 4B). D-PR_24 and D-PR_48 were well separated from the other groups. They are negatively represented by all the variables selected. D-PR_RT and D-PR_72 were discriminated by fructose and dietary fibers variables, while the digesta of the two PRs produced with the use of SD (D-PR_48(SD) and D-PR_48+SD) clustered majorly for all the other variables, in particular, for total FAA and total peptides together with lactic acid and sugars.

## 4. Discussion

Because of the poor digestibility found in the consumption of baker’s yeast products [7,45,46], PR along with other bakery goods needs efforts for possible nutritional improvement. PR can be produced non-conventionally through sourdough fermentation to enhance its compositional, sensory and nutritional properties [47]. In this study, we combined biga and sourdough as two types of baking biotechnologies that are usually used independently to improve digestibility. As the duration of fermentation is known as one of the most important parameters that affect baked goods digestion [9], we considered the effect of different biga fermentation times (24, 48 and 72 h) on PRs digestibility. On the other hand, the positive effects of sourdough fermentation on the digestibility of bakery goods were already demonstrated in both in vitro [9] and in vivo studies [45].

Under our experimental design, the PR made with sourdough-biga fermentation showed the best nutritional profile among the six investigated samples, in agreement with literature dealing with different sourdough baked goods [48,49,50]. In fact, fermentation for 48 h with the biga including sourdough (PR_48(SD)) had the capability to improve the starch digestibility, lowering the pGI of 10.4% compared with the no pre-fermented reference (PR_RT). It can be hypothesized that the higher release of organic acids in PR_48(SD), which is attributable to lactic acid fermentation, had an effect on glucose disposal and on enzyme activity inhibition [46,51] and improved the formation of starch-gluten complex interactions that reduces the bioavailability of starch [52]. The highest content of glucose was released in PR_48(SD) and PR_72 through amylase activities in wheat sourdough and due to bacterial fermentation [53,54]. However, among PRs made with biga, PR_48(SD) and PR_72 had the highest abundance of maltose, which can be attributed to the inhibition of maltogenic amylases by the strong biological acidification (PR_48(SD)) or to the more pronounced hydrolysis of starch by the long biga fermentation (PR_72). Indeed, maltogenic amylases are inhibited at a pH lower than 4.5, while glucoamylases continue their starch and maltodextrin hydrolysis activities that release glucose [54].

Fructose in PR_48(SD) was almost totally converted to mannitol and, although milder, this effect was found also in PR_48+SD. This can be due to the heterofermentative LABs isolated in the SD (e.g., *F. sanfranciscensis*) [55]. The protein digestibility of the control sample made without the use of biga (PR_RT) and with biga including sourdough (PR_48(SD)) was similar as demonstrated by the IVPD index. This could be attributed to the formulation with only wheat flour in the sourdough, instead of the cereal-legume blend (wheat, rice, soy) [56]. Furthermore, such IVPD similarity can depend on the similar content of phytic acid found in the six different PRs. In fact, protein digestibility is affected by the presence of phytic acid and phytates that compromise digestion, decreasing protein solubility or making proteins resistant to proteolytic digestion [57].

As the IVPD analysis did not highlight differences among PRs produced, the EAA index, BV index, PER and NI nutritional indexes were deeply investigated to identify if the production conditions could enhance the protein quality. While BV and EAA indexes were similar among the six PRs, also considering the quantity variations in the protein portion [58], the fermentation conditions of PR_48(SD) and PR_72 led to increased NI compared to the other PRs. Moreover, the PER which reflects the capacity of a protein to support body weight gain [58] increased only when sourdough was combined with yeasted biga ((PR_48(SD)) while it was not affected by the long biga fermentation time. In order to confirm the higher protein quality of PR_48(SD), peptides, FAA and their derivatives were also analyzed. The general increase in peptides and FAA in PR_48(SD) and PR_72 can be linked to fermentation conditions that better promoted the activation of endogenous proteases and increased the solubility of gluten proteins making them labile to enzymatic degradation [54].

The inclusion of sourdough in biga (PR_48(SD)) allowed a significant increase in restrictive EAA, with the only exception of Lys and Thr which could be used as a growing substrate by fermenting microorganisms [59]. The consumption of EAA-enriched foods can increase the quality and quantity of dietary amino acids through a complementary effect of other sources to meet human dietary requirements [60]. GABA is of utmost importance for its diuretic and hypotensive capacity and for its function as a brain sympathetic inhibitory neurotransmitter [61]. A higher concentration of GABA was found in the PR made with biga including sourdough (PR_48(SD)). This positive effect was previously demonstrated in other baked goods, such as breads, where sourdough fermentation enhanced GABA levels [62,63].

Aiming at investigating macronutrient fate along the GI tract and their changes to determine manufacturing parameters that enhance the digestibility of PRs produced with biga, static in vitro digestion was performed. The control sample, PR_RT, was digested under the same conditions.

As the contribution of the gastric phase to the overall digestion of glucose-based polysaccharides is negligible [64,65] and, when plant matrices are digested, FAA present at the end of the gastric digestion account only for 1–2% of the total [66], the digestibility of proteins and carbohydrates was assessed after the small intestinal phase. To the best of our knowledge, this is the first time that such kind of investigation has been conducted for PRs.

The monitoring of simple sugars after dialysis was conducted to assess the glycemic response and to extend our findings on the starch digestibility of PRs. The generation of glucose in D-PR_48(SD) after 3 h of small intestinal dialysis was 48.5% higher than D-PR_RT. This finding is coherent with pGI data and agreed with previous studies on polysaccharides matrices which reported an analogous trend [64]. Therefore, a structural modification of starch or a delay of the glycemic peak could underlie the lower monomer uptake found in PR produced with sourdough-biga [67]. In PR_48(SD), about 50% of the original mannitol was found at the end of the in vitro intestinal digestion (D-PR_48(SD)), while the 70% persisted in the digesta of PR produced by adding sourdough only in the final dough (D-PR_48+SD). Despite this, the mannitol content after in vitro digestion was about five times higher in D-PR_48(SD) compared to D-PR_48+SD. Therefore, it can be hypothesized that the intake of PR_48(SD) can provide a larger quantity of dietary mannitol which acts as a prebiotic in the large intestine, supporting the synthesis (through the fermentation by colon microbiota) of compounds linked to the metabolic health, such as butyrate and propionate [68].

The protein digestibility was assessed by evaluating the soluble digesta nitrogen fraction from peptides and FAA. The OPA method highlighted the higher total peptide content in the digesta of the PR produced with sourdough (D-PR_48(SD)) compared to that of PRs manufactured with baker’s yeast biga. Since the total peptide content did not consider the hydrophobic variations that could be present in the digesta, the peptide release was studied also by using the RP-FPLC analysis. The digesta of PR_48(SD) showed reduced bioavailability of hydrophilic peptides (visible in the first elution step), which can probably be attributed to easier absorption at the small intestine level as a result of consumption of food that was pre-digested during the fermentation process [69].

The inclusion of sourdough enhanced the levels of total FAAs at small intestinal levels, which are typically linked to improved overall health [70].

The digesta analysis showed a positive effect of biga sourdough (D-PR_48(SD)), which increased the level of total FAAs compared to the reference (D-PR_RT). In contrast to what was found in the non-digested counterparts (PRs), after intestinal digestion in vitro, the addition of sourdough in the final dough (D-PR_48+SD) had a similar impact as the inclusion of sourdough in biga (D-PR_48(SD)) in increasing total FAA. Leu and Arg were the most abundant FAA at the end of PR_48(SD) digestion (D-PR_48(SD)), followed by Phe and Tyr [66].

## 5. Conclusions

The use of a biga including sourdough for the manufacturing of PRs resulted in improved carbohydrate digestibility and protein quality after in vitro digestion. The combination of two bakery biotechnologies, SD and biga fermentation, had the capability to reduce the pGI and the glucose absorption and to improve the generation of the protein-related health-promoting compounds and the protein quality indexes. Although the digestibility of baked goods is also related to satiety and gastrointestinal symptoms after consumption [45], such data support the idea that the adoption of a sourdough fermented biga in PR manufacturing can not only enhance its digestibility but also confer added value in the intake of EAA and peptides. Future in vivo trials will be needed to elucidate more the complete mechanism behind the digestibility of PR produced with such a process.

## Figures and Tables

**Table 1:** Pinsa Romana formulas. Ingredients are reported as % final weight (*w*/*w*).

Ingredients (%, *w*/*w*)		PR_24	PR_48	PR_48(SD)	PR_48+SD	PR_72	PR_RT
Pinsa Romana dough							
Wheat flour		-	-	-	-	-	50
Rice flour		-	-	-	-	-	4
Soy flour		-	-	-	-	-	1.5
Biga *		88	88	90	73	88	-
	Wheat flour	51	51	49	42	51	-
	Rice flour	4	4	4.5	4	4	-
	Soy flour	1.5	1.5	1.5	1.5	1.5	-
	Water	31	31	29	25	31	-
	Baker’s yeast	<1	<1	<1	<1	<1	-
	Sourdough	-	-	5	-	-	-
Water		10	10	8	5	10	42
Baker’s yeast		-	-	<1	<1	-	<1
Sourdough		-	-	-	19	-	-
Olive oil		1.5	1.5	1.5	1.5	1.5	1.5
Salt		1	1	1	1	1	1

* Biga were fermented at 16 °C for 24 h (PR_24), 48 h (PR_48, PR_48(SD) or PR_48+SD) and 72 h (PR_72). PR_24, Pinsa Romana made with biga fermented for 24 h with baker’s yeast; PR_48, Pinsa Romana made with biga fermented for 48 h with baker’s yeast; PR_72, Pinsa Romana made with biga fermented for 72 h with baker’s yeast; PR_48(SD), Pinsa Romana made with biga fermented for 48 h with baker’s yeast and sourdough; PR_48+SD, Pinsa Romana made with biga fermented for 48 h with baker’s yeast, and including sourdough in the final dough. PR_RT, Pinsa Romana made without biga and sourdough (control sample).

**Table 2:** Nutritional values of six different Pinsa Romana. PR_24, Pinsa Romana made with biga fermented for 24 h with baker’s yeast; PR_48, Pinsa Romana made with biga fermented for 48 h with baker’s yeast; PR_72, Pinsa Romana made with biga fermented for 72 h with baker’s yeast; PR_48(SD), Pinsa Romana made with biga fermented for 48 h with baker’s yeast and sourdough; PR_48+SD, Pinsa Romana made with biga fermented for 48 h with baker’s yeast, and including sourdough in the final dough. PR_RT, Pinsa Romana made without biga and sourdough (control sample).

	PR_24	PR_48	PR_48(SD)	PR_48+SD	PR_72	PR_RT
Moisture (%)	36.5	34.3	31.5	32.5	33.4	35.5
Total Fat (g/100 g)	5.7	5.6	5.4	5.9	5.5	5.8
Total Carbohydrate (g/100 g)	75.8	76.0	77.4	76.8	76.9	76.8
Ash (g/100 g)	2.5	2.7	2.4	2.5	2.4	2.5
Protein (g/100 g)	16.0	15.8	14.8	14.8	15.2	14.9
Energy value (Kcal/100 g)	413	406	411	417	412	411

**Table 3:** Biochemical and nutritional characteristics of the six Pinsa Romana. pH, total titratable (TTA) (mL of NaOH/pH 8.3), phytic acid (g/100 g), in vitro protein digestibility (IVPD%) and predicted glycemic index (pGI). In the same column, values with different superscript letters differ significantly (*p* < 0.05) based on one-way ANOVA (Tukey–Kramer). The data are the means of three independent analyses ± standard deviations (*n* = 3). PR_24, Pinsa Romana made with biga fermented for 24 h with baker’s yeast; PR_48, Pinsa Romana made with biga fermented for 48 h with baker’s yeast; PR_72, Pinsa Romana made with biga fermented for 72 h with baker’s yeast; PR_48(SD), Pinsa Romana made with biga fermented for 48 h with baker’s yeast and sourdough; PR_48+SD, Pinsa Romana made with biga fermented for 48 h with baker’s yeast, and including sourdough in the final dough. PR_RT, Pinsa Romana made without biga and sourdough (control sample).

	pH	TTA	Phytic Acid	IVPD	pGI
PR_24	5.61 ± 0.01 ^a^	2.9 ± 0.1 ^d^	0.12 ± 0.02 ^a^	62.9 ± 0.4 ^a^	68.8 ± 0.3 ^a^
PR_48	5.48 ± 0.02 ^b^	3.3 ± 0.1 ^c^	0.11 ± 0.02 ^a^	63.0 ± 0.8 ^a^	67.8 ± 0.8 ^a^
PR_48(SD)	4.27 ± 0.02 ^d^	8.4 ± 0.3 ^a^	0.09 ± 0.01 ^a^	60.3 ± 0.4 ^a^	63.9 ± 0.1 ^b^
PR_48+SD	4.90 ± 0.02 ^c^	5.1 ± 0.1 ^b^	0.09 ± 0.02 ^a^	60.6 ± 1.1 ^a^	68.0 ± 1.1 ^a^
PR_72	5.41 ± 0.04 ^b^	3.1 ± 0.1 ^cd^	0.11 ± 0.01 ^a^	62.7 ± 1.9 ^a^	68.5 ± 0.4 ^a^
PR_RT	5.60 ± 0.03 ^a^	2.3 ± 0.1 ^e^	0.12 ± 0.02 ^a^	62.2 ± 1.4 ^a^	71.3 ± 1.8 ^a^

**Table 4:** Nutritional indexes of six different Pinsa Romana: essential amino acid (EAA) index, biological value (BV) index, protein efficiency ratio (PER), Nutritional Index (NI) and sequence of limiting EAA. Values in the same row with different superscript letters differ significantly (*p* < 0.05) based on one-way ANOVA (Tukey–Kramer). The data are the means of three independent analyses ± standard deviations (*n* = 3). PR_24, Pinsa Romana made with biga fermented for 24 h with baker’s yeast; PR_48, Pinsa Romana made with biga fermented for 48 h with baker’s yeast; PR_72, Pinsa Romana made with biga fermented for 72 h with baker’s yeast; PR_48(SD), Pinsa Romana made with biga fermented for 48 h with baker’s yeast and sourdough; PR_48+SD, Pinsa Romana made with biga fermented for 48 h with baker’s yeast and including sourdough in the final dough. PR_RT, Pinsa Romana made without biga and sourdough (control sample).

	PR_24	PR_48	PR_48(SD)	PR_48+SD	PR_72	PR_RT
EAA index	68.97 ± 3.73 ^a^	68.99 ± 4.13 ^a^	72.61 ± 3.50 ^a^	71.55 ± 4.44 ^a^	75.26 ± 4.87 ^a^	75.85 ± 5.70 ^a^
BV index	63.48 ± 4.11 ^a^	63.50 ± 3.82 ^a^	67.44 ± 4.22 ^a^	66.29 ± 4.75 ^a^	70.33 ± 4.26 ^a^	70.98 ± 3.98 ^a^
PER	22.36 ± 1.71 ^bc^	24.69 ± 2.11 ^b^	30.33 ± 1.54 ^a^	23.32 ± 1.05 ^bc^	24.37 ± 1.61 ^b^	19.19 ± 1.29 ^c^
NI	2.91 ± 0.13 ^b^	2.86 ± 0.12 ^b^	3.87 ± 0.27 ^a^	3.03 ± 0.12 ^b^	3.60 ± 0.17 ^a^	2.77 ± 0.19 ^b^
Sequence of Limiting EAA	Leu	Lys	Thr	Lys	His	Met
	His	Trp	Lys	Thr	Lys	Leu
	Trp	Leu	His	His	Thr	His

**Table 5:** Biochemical and nutritional characteristics of Pinsa Romana before and after small intestinal in vitro digestion; lactic and acetic acid (mM), glucose (mM), fructose (mM), mannitol (mM), maltose (mM), peptides (g/Kg), resistant starch (RS, g/100 g d.w.), and dietary fibers (% d.m.). Values in the same column with different superscript letters differ significantly (*p* < 0.05) based on one-way ANOVA (Tukey–Kramer). The data are the means of three independent analyses ± standard deviations (*n* = 3). LOQ = limit of quantification. PR_24, Pinsa Romana made with biga fermented for 24 h with baker’s yeast; PR_48, Pinsa Romana made with biga fermented for 48 h with baker’s yeast; PR_72, Pinsa Romana made with biga fermented for 72 h with baker’s yeast; PR_48(SD), Pinsa Romana made with biga fermented for 48 h with baker’s yeast and sourdough; PR_48+SD, Pinsa Romana made with biga fermented for 48 h with baker’s yeast, and including sourdough in the final dough. PR_RT, Pinsa Romana made without biga and sourdough (control sample). “D-” prefix means digested.

	Lactic Acid	Acetic Acid	Glucose	Fructose	Mannitol	Maltose	Peptides	RS	Dietary Fibers
Pinsa Romana									
PR_24	2.9 ± 0.1 ^c^	<LOQ	11.0 ± 0.1 ^d^	6.7 ± 0.1 ^b^	<LOQ	46.7 ± 0.1 ^c^	9.3 ± 0.1 ^d^	1.1 ± 0.1 ^a^	14.4 ± 0.6 ^a^
PR_48	2.5 ± 0.6 ^c^	<LOQ	10.0 ± 0.1 ^d^	5.8 ± 0.1 ^b^	<LOQ	35.4 ± 0.1 ^e^	9.5 ± 0.1 ^d^	0.8 ± 0.2 ^ab^	15.4 ± 1.7 ^a^
PR_48(SD)	102.1 ±0.1 ^a^	27.0 ± 0.1 ^a^	32.7 ± 0.1 ^a^	2.3 ± 0.1 ^d^	41.8 ± 0.1 ^a^	60.6 ± 0.1 ^b^	12.7 ± 0.2 ^a^	0.9 ± 0.1 ^ab^	14.8 ± 1.3 ^a^
PR_48+SD	35.7 ± 0.1 ^b^	14.7 ± 0.3 ^b^	17.6 ± 0.1 ^c^	4.5 ± 0.1 ^c^	6.2 ± 0.1 ^b^	43.9 ± 0.1 ^d^	12.0 ± 0.1 ^b^	0.7 ± 0.1 ^b^	14.8 ± 0.8 ^a^
PR_72	2.3 ± 0.1 ^c^	1.2 ± 0.3 ^c^	21.1 ± 0.1 ^b^	14.0 ± 0.1 ^a^	<LOQ	61.0 ± 0.1 ^b^	11.4 ± 0.1 ^c^	0.8 ± 0.1 ^ab^	13.7 ± 0.7 ^a^
PR_RT	2.6 ± 0.2 ^c^	<LOQ	3.1 ± 0.3 ^e^	6.9 ± 0.1 ^b^	<LOQ	74.7 ± 0.1 ^a^	9.1 ± 0.2 ^d^	1.0 ± 0.1 ^ab^	14.2 ± 1.7 ^a^
Digested (D) Pinsa Romana									
D-PR_24	<LOQ	<LOQ	144.4 ± 12.1 ^ab^	13.3 ± 0.1 ^b^	0.9 ± 0.6 ^c^	699.6 ± 23.7 ^a^	145.2 ± 7.2 ^c^	0.7 ± 0.1 ^a^	15.3 ± 1.7 ^a^
D-PR_48	<LOQ	<LOQ	137.3 ± 8.2 ^b^	<LOQ	0.1 ± 0.1 ^c^	722.9 ± 35.2 ^a^	159.4 ± 6.9 ^bc^	0.8 ± 0.1 ^a^	14.0 ± 0.2 ^a^
D-PR_48(SD)	42.0 ± 3.0 ^a^	<LOQ	188.5 ± 3.3 ^a^	<LOQ	21.0 ± 2.1 ^a^	741.2 ± 15.2 ^a^	201.2 ± 8.1 ^a^	0.9 ± 0.1 ^a^	13.4 ± 0.9 ^a^
D-PR_48+SD	18.7 ± 0.6 ^b^	<LOQ	149.7 ± 15.0 ^ab^	<LOQ	4.4 ± 2.3 ^b^	728.1 ± 55.1 ^a^	183.1 ± 1.5 ^ab^	0.9 ± 0.1 ^a^	16.6 ± 0.1 ^a^
D-PR_72	<LOQ	<LOQ	146.1 ± 4.5 ^ab^	15.2 ± 1.1 ^a^	<LOQ	709.9 ± 45.3 ^a^	165.8 ± 1.3 ^bc^	0.9 ± 0.1 ^a^	16.0 ± 0.7 ^a^
D-PR_RT	<LOQ	<LOQ	126.9 ± 22.7 ^b^	12.9 ± 0.2 ^b^	<LOQ	665.8 ± 41.4 ^a^	146.9 ± 2.3 ^c^	0.9 ± 0.1 ^a^	16.6 ± 1.1 ^a^

**Table 6:** Area under the curve (mAU·min) of peptides at different CH_3_CN gradient in Pinsa Romana before and after digestion process expressed as percentage (%) on the total area. PR_24, Pinsa Romana made with biga fermented for 24 h with baker’s yeast; PR_48, Pinsa Romana made with biga fermented for 48 h with baker’s yeast; PR_72, Pinsa Romana made with biga fermented for 72 h with baker’s yeast; PR_48(SD), Pinsa Romana made with biga fermented for 48 h with baker’s yeast and sourdough; PR_48+SD, Pinsa Romana made with biga fermented for 48 h with baker’s yeast, and including sourdough in the final dough. PR_RT, Pinsa Romana made without biga and sourdough (control sample). “D-” prefix means digested. AUC, area under the curve (mAU·min).

	CH_3_CN < 19%	19% < CH_3_CN < 41%	CH_3_CN > 41%	Total AUC
Pinsa Romana				
PR_24	12.9	87.1	0	2511.3
PR_48	17.5	82.6	0	2659.0
PR_48(SD)	19.1	80.9	0	3693.0
PR_48+SD	5.8	94.2	0	3273.8
PR_72	22.6	77.4	0	2713.8
PR_RT	18.1	81.8	0	2196.9
Digested (D) Pinsa Romana				
D-PR_24	67.5	17.5	15.1	3307.5
D-PR_48	63.2	20.3	16.5	3594.8
D-PR_48(SD)	58.8	28.9	12.4	3478.6
D-PR_48+SD	66.5	18.4	15.1	3185.7
D-PR_72	66.0	21.5	12.5	3518.8
D-PR_RT	66.5	18.4	15.1	3611.1

## Data Availability

The data presented in this study are available on request from the corresponding author. The data are not publicly available due to legal issues.

## Supplementary Materials

The following supporting information can be downloaded at: https://www.mdpi.com/article/10.3390/nu15132958/s1, Table S1: List and description of biga and Pinsa Romana used in this study; Table S2: Characterization of biga and sourdough; Table S3: Free amino acids profile of Pinsa Romana before (mg/kg) and after (mg/L) digestion.
