# Supplementary material for: Sourdough “Biga” Fermentation Improves the Digestibility of Pizza Pinsa Romana: An Investigation through a Simulated Static In Vitro Model
Source: Nutrients. 2023 Jun 29;15(13):2958. doi: 10.3390/nu15132958 (PMC10346773; doi:10.3390/nu15132958)
Supplement: Supplementary file 1 [file nutrients-15-02958-s001.zip › nutrients-2462173-supplementary.pdf]

Table S1. List and description of biga and Pinsa Romana used in this study.

BIGA		N	PINSA ROMANA		
**Code**	**Conditions**	**N**	**Type of process**	**Code**	
**B_24**	Ingredients: flour (wheat, rice, and soy), water, baker's yeast. Fermentation, 24 h at 16 °C.	**1**	Indirect	**PR_24**	Ingredients: B_24, water, baker's yeast, olive oil, and salt. Leavened for 2 h at 24 °C; 1.5 h processing and resting.
**$B\_48_{(1)}$**	Ingredients: flour (wheat, rice, and soy), water, baker's yeast. Fermentation, 48 h at 16 °C.	**2**	Indirect	**PR_48**	Ingredients: $B\_48_{(1)}$, water, baker's yeast, olive oil, and salt. Leavened for 2 h at 24 °C; 1.5 h processing and resting.
**$B\_48_{(2)}$**	Ingredients: flour (wheat, rice, and soy), water, baker's yeast. Fermentation, 48 h at 16 °C.	**3**	Indirect	**PR_48+SD**	Ingredients: $B\_48_{(2)}$, water, sourdough, baker's yeast, olive oil and salt. Leavened for 2 h at 24 °C; 1.5 h processing and resting.
**B_48(SD)**	Ingredients: flour (wheat, rice, and soy), water, sourdough, baker's yeast. Fermentation, 48 h at 16 °C.	**4**	Indirect	**PR_48(SD)**	Ingredients: B_48(SD), water, baker's yeast, olive oil, and salt. Leavened for 2 h at 24 °C; 1.5 h processing and resting.
**B_72**	Ingredients: flour (wheat, rice, and soy), water, baker's yeast. Fermentation, 72 h at 16 °C.	**5**	Indirect	**PR_72**	Ingredients: B_72, water, baker's yeast, olive oil, and salt. Leavened for 2 h at 24 °C; 1.5 h processing and resting.
**-**	**-**	**6**	Direct	**PR_RT**	Ingredients: flour (wheat, rice and soy), water, baker's yeast, olive oil and salt. Leavened for 2 h at 24 °C; 1.5 h processing and resting.

$B\_48_{(1)}$ and $B\_48_{(2)}$ biga have the same fermentation conditions and kind of raw ingredients, while they differ only for the percentage (weight/final weight) of each raw ingredient (see Table 1).

Table S2. Characterization of biga and sourdough.

	pH	LAB (Log CFU/g)	Yeast (Log CFU/g)
B_24	5.48 ± 0.04	6.40 ± 0.21	7.72 ± 0.14
B_48$_{(1)}$	5.41 ± 0.02	6.35 ± 0.17	8.00 ± 0.02
B_48$_{(2)}$	5.42 ± 0.01	6.20 ± 0.25	7.93 ± 0.02
B_48(SD)	3.94 ± 0.01	8.89 ± 0.13	7.22 ± 0.17
B_72	5.55 ± 0.04	7.91 ± 0.06	7.04 ± 0.02
CT_D	5.42 ± 0.02	< 5	8.04 ± 0.04
SD	3.78 ± 0.03	8.06 ± 0.01	7.33 ± 0.13

The data are the means of three independent analysis ± standard deviations (n =3). SD, sourdough; B_24, biga fermented for 24 h with baker´s yeast; B_48$_{(1)}$, biga fermented 48 h with baker´s yeast; B_48$_{(2)}$, biga fermented 48 h with baker´s yeast; B_48(SD), biga fermented for 48 h with baker´s yeast and sourdough; B_72, biga fermented for 72 h with baker's yeast; CT_D, control dough made without biga and sourdough.

Table S3. Free amino acids profile of Pinsa Romana before (mg/kg) and after (mg/L) digestion**.**

	Pinsa Romana					
	PR_24	PR_48	PR_48(SD)	PR_48+SD	PR_72	PR_RT
Asp	14.35 ± 0.21[d]	13.43 ± 0.20[d]	95.45 ± 1.41[a]	39.09 ± 0.58[c]	57.23 ± 0.84[b]	13.82 ± 0.20[d]
Thr	4.76 ± 0.13[c]	4.16 ± 0.11[d]	4.71 ± 0.13[c]	4.35 ± 0.12[cd]	7.87 ± 0.21[b]	10.11 ± 0.27[a]
Ser	3.12 ± 0.08[d]	2.06 ± 0.05[e]	12.56 ± 0.31[a]	3.79 ± 0.09[c]	3.98 ± 0.10[c]	7.66 ± 0.19[b]
Glu	18.93 ± 0.64[d]	13.32 ± 0.45[e]	69.43 ± 2.34[a]	31.05 ± 1.05[b]	24.55 ± 0.83[c]	19.83 ± 0.67[d]
Gly	12.86 ± 0.29[b]	10.46 ± 0.24[c]	1.10 ± 0.03[d]	12.40 ± 0.28[b]	22.80 ± 0.52[a]	22.83 ± 0.52[a]
Ala	10.95 ± 0.24[f]	13.6 ± 0.30[e]	19.56 ± 0.43[c]	16.07 ± 0.35[d]	27.95 ± 0.62[b]	31.41 ± 0.69[a]
Val	11.03 ± 0.30[de]	10.04 ± 0.27[e]	21.85 ± 0.59[a]	11.95 ± 0.32[d]	19.20 ± 0.52[b]	14.79 ± 0.40[c]
Cys	24.36 ± 0.99[a]	20.30 ± 0.83[bc]	21.27 ± 0.87[bc]	19.00 ± 0.77[c]	21.49 ± 0.88[b]	25.01 ± 1.02[a]
Met	4.46 ± 0.15[d]	4.02 ± 0.14[d]	8.80 ± 0.30[a]	5.58 ± 0.19[c]	6.24 ± 0.21[b]	2.47 ± 0.08[e]
Ile	10.92 ± 0.31[d]	10.34 ± 0.30[d]	17.80 ± 0.51[a]	12.54 ± 0.36[c]	14.48 ± 0.42[b]	11.02 ± 0.32[d]
Leu	2.38 ± 0.08[d]	1.86 ± 0.06[d]	37.28 ± 1.28[a]	5.94 ± 0.20[c]	12.83 ± 0.44[b]	2.41 ± 0.08[d]
Tyr	1.67 ± 0.07[e]	0.90 ± 0.04[e]	21.72 ± 0.87[a]	10.53 ± 0.42[c]	19.18 ± 0.77[b]	5.31 ± 0.21[d]
Phe	1.50 ± 0.06[d]	1.39 ± 0.06[d]	28.25 ± 1.13[a]	6.13 ± 0.25[c]	14.40 ± 0.58[b]	2.72 ± 0.11[d]
GABA	7.53 ± 0.11[e]	4.58 ± 0.07[f]	66.92 ± 0.97[a]	45.65 ± 0.66[c]	59.67 ± 0.87[b]	32.87 ± 0.48[d]
Orn	2.53 ± 0.08[c]	3.46 ± 0.11[b]	3.93 ± 0.13[a]	3.92 ± 0.13[a]	3.49 ± 0.12[b]	0.53 ± 0.02[d]
Lys	7.33 ± 0.18[c]	4.25 ± 0.11[e]	5.01 ± 0.13[d]	4.49 ± 0.11[de]	10.02 ± 0.25[b]	12.34 ± 0.31[a]

Table S3. *Cont.*

	PR_24	PR_48	PR_48(SD)	PR_48+SD	PR_72	PR_RT
His	1.15 ± 0.03^{c}	1.64 ± 0.05^{b}	1.22 ± 0.04^{c}	1.19 ± 0.04^{c}	2.05 ± 0.06^{a}	1.58 ± 0.05^{b}
Trp	0.29 ± 0.01^{d}	0.02 ± 0.01^{d}	26.88 ± 1.02^{a}	10.59 ± 0.40^{c}	20.56 ± 0.78^{b}	20.22 ± 0.76^{b}
Arg	35.69 ± 0.88^{d}	23.03 ± 0.57^{e}	72.71 ± 1.79^{a}	41.47 ± 1.02^{c}	45.38 ± 1.12^{b}	34.33 ± 0.84^{d}
Pro	14.39 ± 0.73^{d}	18.33 ± 0.93^{c}	20.68 ± 1.05^{c}	25.78 ± 1.31^{b}	32.20 ± 1.64^{a}	24.04 ± 1.22^{b}
Total	190.22 ± 5.16^{d}	161.19 ± 4.35^{e}	557.14 ± 14.72^{a}	311.49 ± 8.16^{c}	425.56 ± 11.29^{b}	295.31 ± 8.03^{c}
			Digested (D) Pinsa Romana			
	D-PR_24	D-PR_48	D-PR_48(SD)	D-PR_48+SD	D-PR_72	D-PR_RT
Asp	12.06 ± 3.14^{b}	11.94 ± 1.04^{b}	22.56 ± 0.89^{a}	22.48 ± 0.66^{a}	20.68 ± 2.25^{a}	14.29 ± 2.11^{b}
Thr	32.32 ± 3.13^{c}	32.29 ± 4.64^{c}	56.41 ± 0.90^{a}	51.77 ± 3.95^{ab}	47.14 ± 3.00^{b}	36.49 ± 2.22^{c}
Ser	40.29 ± 1.63^{d}	38.38 ± 4.87^{d}	76.15 ± 0.61^{a}	64.69 ± 7.58^{ab}	59.37 ± 4.54^{bc}	47.10 ± 4.59^{cd}
Glu	46.02 ± 4.91^{d}	47.79 ± 6.29^{cd}	97.97 ± 0.99^{a}	78.01 ± 5.59^{b}	74.41 ± 2.77^{b}	59.30 ± 4.73^{c}
Gly	15.31 ± 0.38^{c}	15.58 ± 2.12^{c}	27.81 ± 0.33^{a}	24.84 ± 2.89^{a}	22.94 ± 1.74^{ab}	18.40 ± 1.63^{bc}
Ala	44.12 ± 4.00^{c}	46.64 ± 5.96^{c}	72.72 ± 3.30^{a}	70.19 ± 6.89^{a}	61.27 ± 3.73^{ab}	48.93 ± 2.50^{bc}
Val	58.25 ± 4.47^{c}	61.91 ± 7.06^{c}	98.88 ± 4.74^{a}	93.85 ± 7.67^{ab}	83.70 ± 3.45^{b}	64.12 ± 3.74^{c}
Cys	16.86 ± 0.49^{c}	19.20 ± 3.74^{bc}	33.94 ± 2.15^{a}	35.04 ± 1.30^{a}	33.17 ± 0.38^{a}	23.51 ± 2.97^{b}
Met	20.70 ± 2.81^{c}	23.78 ± 3.14^{bc}	34.23 ± 3.13^{a}	34.96 ± 1.02^{a}	29.88 ± 0.49^{ab}	22.05 ± 0.95^{c}

Table S3. *Cont.*

	D-PR_24	D-PR_48	D-PR_48(SD)	D-PR_48+SD	D-PR_72	D-PR_RT
Ile	53.02 ± 3.24^{c}	54.20 ± 6.89^{c}	87.40 ± 3.51^{a}	81.57 ± 7.22^{ab}	72.89 ± 2.33^{b}	57.61 ± 2.38^{c}
Leu	105.81 ± 9.30^{c}	116.11 ± 16.98^{bc}	158.83 ± 7.84^{a}	155.39 ± 12.14^{a}	137.75 ± 1.38^{ab}	111.67 ± 0.01^{bc}
Tyr	67.14 ± 6.32^{c}	76.50 ± 10.40^{bc}	107.24 ± 6.75^{a}	104.90 ± 5.77^{a}	92.52 ± 0.69^{ab}	72.59 ± 0.03^{c}
Phe	70.86 ± 9.15^{c}	78.96 ± 10.90^{bc}	109.01 ± 6.54^{a}	109.95 ± 4.00^{a}	96.69 ± 0.01^{ab}	74.39 ± 0.63^{c}
GABA	1.63 ± 0.71^{a}	2.25 ± 0.30^{a}	3.06 ± 0.71^{a}	2.26 ± 1.45^{a}	2.78 ± 0.01^{a}	1.08 ± 0.30^{a}
Orn	0.69 ± 0.04^{c}	1.57 ± 0.33^{ab}	2.19 ± 0.03^{a}	2.00 ± 0.25^{ab}	1.39 ± 0.42^{b}	1.52 ± 0.12^{ab}
Lys	49.28 ± 4.94^{d}	50.75 ± 5.88^{d}	77.37 ± 4.84^{a}	75.07 ± 4.26^{ab}	64.49 ± 0.42^{bc}	53.17 ± 1.55^{cd}
His	25.18 ± 2.06^{c}	26.44 ± 3.27^{c}	42.61 ± 2.09^{a}	39.48 ± 2.30^{a}	33.75 ± 0.81^{b}	27.68 ± 0.86^{c}
Trp	26.79 ± 3.00^{c}	27.07 ± 3.28^{c}	41.59 ± 2.37^{a}	40.51 ± 2.42^{ab}	35.19 ± 0.44^{b}	27.01 ± 0.80^{c}
Arg	72.52 ± 8.88^{c}	81.11 ± 9.47^{bc}	114.04 ± 7.43^{a}	112.21 ± 4.07^{a}	92.06 ± 1.05^{b}	75.45 ± 0.12^{bc}
Pro	4.53 ± 0.13^{d}	4.42 ± 0.29^{d}	11.31 ± 0.97^{a}	6.71 ± 0.33^{b}	6.07 ± 0.47^{bc}	4.95 ± 0.43^{cd}
Total	763.38 ± 71.96^{c}	816.89 ± 110.84^{c}	1275.30 ± 51.92^{a}	1205.88 ± 81.57^{a}	1068.14 ± 26.98^{b}	841.31 ± 32.30^{c}

Values in the same row with different superscript letters differ significantly ($p < 0.05$) based on one-way ANOVA (Tuckey-Kramer). The data are the means of three independent analysis ± standard deviations (n =3). PR_24, Pinsa Romana made with biga fermented for 24 h with baker´s yeast; PR_48, Pinsa Romana made with biga fermented for 48 h with baker´s yeast; PR_72, Pinsa Romana made with biga fermented for 72 h with baker´s yeast; PR_48(SD), Pinsa Romana made with biga fermented for 48 h with baker´s yeast and

sourdough; PR_48+SD, Pinsa Romana made with biga fermented for 48 h with baker´s yeast, and including sourdough in the final dough. PR_RT, Pinsa Romana made without biga and sourdough (reference thesis).
